# Validation of a Low-Cost Video-Based Competency Score For Assessing Safe Falling Movement Patterns in Older Adults

**DOI:** 10.1093/geroni/igaf122.1618

**Published:** 2025-12-31

**Authors:** Lingjun Chen, Tobia Zanotto, James Fang, Joohyun Lee, Neil Alexander, Jacob Sosnoff

**Affiliations:** University of Kansas Medical Center, Kansas City, Kansas, United States; University of Kansas Medical Center, Kansas City, Kansas, United States; University of Kansas Medical Center, Kansas City, Kansas, United States; University of Kansas Medical center, Kansas City, Kansas, United States; University of Michigan, Ann Arbor, Michigan, United States; University of Kansas Medical Center, Kansas City, Kansas, United States

## Abstract

Here is growing interest in enabling older adults with skills to reduce fall-related injury. One approach involves teaching older adults safely falling movements, such as tuck and roll, to minimize fall-related injury risk. However, the current methods for evaluating fall movement patterns require biomechanical assessments and are not clinical feasible. To address this gap, Safe Falling Competency Score was developed, a standardized and low-cost approach to assess key evidence-based safe falling movement patterns: 1) active attempt of squatting (to lower the center of mass); 2) trunk rotation and rolling (to reduce vertical impact); 3) appropriate upper limb use (to avoid outstretched hand landing); 4) active and successful chin tuck (to prevent head impact). This study validated the competency score using 264 video-recorded experimental falls from 24 older adults at risk for falls (72.53 ± 5.39 years), who completed the pre- and post-training assessments in a Safely Falling Training Project (R21AG07892). Two independent reviewers scored the falls, with discrepancies resolved via discussion (excellent inter-rater reliability, ICC = 0.713). Head and hip acceleration of each fall were extracted from motion capture data using custom MatLab script. Spearman’s correlation revealed that higher competency scores were associated with the reduced head (ρ = -0.3705, p < 0.0001) and hip accelerations (ρ = -0.293, p < 0.0001), supporting the score’s validity. These findings suggest the Safe Fall Competency Score provides a reliable and valid tool for clinicians to assess fall movement patterns and fall-related injury risk.

